# Inhibitors Targeting Multiple Janus Kinases From *Zanthoxylum simulans* Mediate Inhibition and Apoptosis Against Gastric Cancer Cells *via* the Estrogen Pathway

**DOI:** 10.3389/fchem.2022.922110

**Published:** 2022-06-06

**Authors:** Yong-Qiang Tian, Dai Hu, Yong-Li Zhang, Jian Zou, Gui-Lin Chen, Ming-Quan Guo

**Affiliations:** ^1^ Department of Pharmacy, Wuhan Hospital of Traditional Chinese Medicine, Third Clinical Medical College of Hubei University of Chinese Medicine, Wuhan, China; ^2^ CAS Key Laboratory of Plant Germplasm Enhancement and Specialty Agriculture, Wuhan Botanical Garden, Chinese Academy of Sciences, Wuhan, China; ^3^ Department of Pharmacy, The Central Hospital of Wuhan, Tongji Medical College, Huazhong University of Science and Technology, Wuhan, China; ^4^ Sino-Africa Joint Research Center, Chinese Academy of Sciences, Wuhan, China; ^5^ Innovation Academy for Drug Discovery and Development, Chinese Academy of Sciences, Shanghai, China

**Keywords:** Janus kinases inhibitors, total quaternary alkaloids (QAs), *Zanthoxylum simulans*, gastric cancer (AGS), anti-proliferative activity, estrogen receptors

## Abstract

Janus kinases (JAKs) play a key role in subtly regulating proliferation, apoptosis, and differentiation of cancer cells, and their inhibitors are actively sought as new drug leads. By developing JAKs based affinity ultrafiltration method coupled with LC/Q-TOF-MS in order to discover selective JAKs inhibitors from total quaternary alkaloids (QAs) from *Zanthoxylum simulans*, peak 19 (Berberine) and peak 21 (Chelerythrine) were revealed to exhibit notable selectivity on JAK1, JAK2, and JAK3 over Tyk2. In addition, Chelerythrine showed stronger inhibitory activity than the positive control (Cerdulatinib) on gastric cancer cells (AGS), while Berberine, with weaker inhibition. Chelerythrine and Berberine also showed obvious inhibition on human hepatocyte cells (LO2). Furthermore, molecular docking analysis revealed their discrepancies due to different interaction bonds and characteristic residues. Quaternary N was proposed as the functional group to enhance the selectivity of JAK1, and some specific moieties towards Asp1021, Leu855, and Leu828 were suggested to increase the selectivity for JAK1, JAK2, and JAK3, respectively. As the most potential inhibitor of JAKs from QAs, Chelerythrine exhibited distinct suppression of adhesion, migration, invasion, and stimulating apoptosis of AGS cells, which was consistent with the significant down-regulation of estrogen receptors (ER-α36, ER-α66, and ER-β1) and Src expression. In conclusion, an efficient screening approach was developed to identify Berberine and Chelerythrine as potential selective candidates from *Zanthoxylum simulans* with significant anti-proliferative activity against gastric carcinoma. As we know, it was the first report to propose an estrogen signal pathway for Chelerythrine in anti-gastric cancer cells (AGS) study. The results supported Chelerythrine inhibitory effects on AGS by not only direct inhibiting JAKs but also down-regulating the estrogen pathway.

## Introduction

It is well known that gastric cancer is often developed from normal gastric mucosa cells involving many risk factors, such as *Helicobacter pylori* infection, infection treatment, environmental factors, etc. (de Martel et al., 2020; Kumar et al., 2020). With high mortality and complex medical treatment, the carcinogenic mechanisms of gastric cancer have been well studied in recent years (Kumar et al., 2020). It is reported that JAK genes in gastric cancer were up-regulated and the kinases were significantly increased compared with normal stomach cells, which indicates that JAKs may play an important role in the development and treatment of gastric cancer (Wang et al., 2017). Including JAK1, JAK2, JAK3, and Tyk2, various JAKs are always activated by cytokines to mediate the gastric cancer cells progression, such as JAK1 kinase by IL-10 family cytokines, JAK2 kinase by G-CSF factor, Tyk2 kinase by cytokine IL-23, respectively (De Velasco et al., 2016; O'Shea et al., 2015). Therefore, JAKs inhibitors could be considered as potential anti-gastric cancer drugs. Signal transducer and activator of transcription (STAT) has seven subtypes, including STAT1, STAT2, STAT3, STAT4, STAT5a, STAT5b, and STAT6, which are activated by different JAKs to play the conflicting roles in promoting or inhibiting the tumor cell proliferation (O’Keefe and Grandis, 2016). Considering the contrary activity by diverse pathways, including JAK1-STAT3, Tyk2-STAT2, JAK3-STAT5, etc. (Wu et al., 2016), the screening of selective inhibitors of JAKs may lead to the discovery of anti-gastric cancer drug candidates with lower side effects.

It is well known that estrogens are mainly responsible for the control of functions of the female reproductive system, which exert their actions by binding to estrogen receptors (ERs, including ERα and ERβ) to regulate gene expression and activate the hormone-related cross-talk signaling cascades (Nathalie and Patricia, 2019). The incidence of gastric carcinoma in men is higher than in women based on epidemiological investigations, which indicate the potential protective effect of estrogen and estrogen receptors (Wang, 2013; Wesołowska et al., 2016). Further studies showed that estrogen played an important role in gastric cancer cells proliferation and invasion (Tang et al., 2017; Ge et al., 2018; Yongle Zhang et al., 2020), which could be mediated *via* the Src signal pathway by estrogen receptors, for example, ER-α36 (Wang et al., 2013; Wang et al., 2020). In addition, evidence suggested that the estrogen-related signal pathway could be regulated by JAK/STAT (Siersbæk et al., 2020; Wu et al., 2020; Hanson et al., 2021). Thus, it is necessary to interpret the regulation of estrogen gene expression when cells were treated with JAK inhibitors.

Belonging to the genus *Zanthoxylum* of the Rutaceae family, *Zanthoxylum simulans* is widely distributed in China according to the record in “Chinese Flora” (Committee of Chinese Flora of Chinese Academy of Sciences, 1997). *Z. simulans* contains a variety of chemical components, such as volatile oil, lignin, flavonoids and alkaloids, which contribute largely to its arresting analgesic, stomachic, antibacterial and roundworm-riding activities (Qi et al., 2015; Tian et al., 2017; Kuo et al., 2021). Wherein, alkaloids from the *Zanthoxylum* genus are considered to be responsible for its notable anti-tumor activities (Lin et al., 2020). In our previous studies, the quaternary alkaloids (QAs) from the *Z. simulans* extracts were found to be the most potential bioactive components of human gastric cancer cells (SGC-7901) (Tian et al., 2017). Therefore, the screening and identification of specific QAs with reasonable activity and selectivity may help with providing anti-gastric cancer candidates or pro-drugs with lower toxicity to the human body as natural medicines.

Traditionally, methods for screening bioactive components from traditional Chinese medicine (TCM) required multi-step chemical isolations and activity-guided experiments, which were recognized to be complicated and time-consuming (Varela and Fernandes, 2020). To this end, affinity ultrafiltration has been applied to rapidly screen potential bioactive compounds in recent years (Chen et al., 2019). In ultrafiltration, key proteins in the development of diseases are selected as the targets and adopted to screen the potential ligands from complex extracts of TCM based on the ligand-target affinity (Qin et al., 2018; Lu et al., 2019). Once active candidates are found, activity analyses are needed to verify the results of ultrafiltration. Hence, affinity ultrafiltration has been widely reported and proved to be sensitive and efficient for selective inhibitor discovery (Bingjie Zhang et al., 2020).

To find the potential ligands from crude QAs targeting to JAKs efficiently, a method of affinity ultrafiltration coupled with LC-ESI-Q-TOF (UF-LC-MS) was developed and adopted in this study. In addition, the most potential alkaloids were employed in the investigation of inhibition, adhesion, migration, and invasion of human normal hepatocytes (LO2) and gastric cancer (AGS) cells. The mechanisms of AGS were elucidated by molecular docking and estrogen-dependent regulations.

## Materials and Methods

### Chemicals and Reagents

Standards of chemicals used in this study, including Chelerythrine (98%, CAS: 3895-92-9), Berberine (98%, CAS: 2086-83-1), Magnoflorine (99%, CAS: 2141-09-5), and Cerdualtinib (98.5%, CAS: 1198300-79-6), were provided by National Institutes for Food and Drug Control (Beijing, China), Chengdu Pufei De Biotech Co., Ltd. (Chengdu, China), Shanghai Macklin Biochemical Co., Ltd. (Shanghai, China) and Target Molecule Corp. (Shanghai, China), respectively. Water for HPLC and LC-MS was prepared from EPED (Nanjing Yeap Esselte Technology Development Co., Nanjing, China). LO2 cell lines and AGS were purchased from Procell Life Science & Technology Co. Ltd. (Wuhan, China), and BeNa Culture Collection (Beijing, China), respectively. Antibiotics added in cell culture media, Penicillin and Streptomycin, were obtained from Sigma-Aldrich Corp. (St. Louis, MO, United States). Roswell Park Memorial Institute (RPMI) 1640, Ham’s F-12K (Kaighn’s) Medium (F-12K), and fetal bovine serum (FBS) were supplied by Gibco Life Technologies (Grand Island, NY, United States). Antibodies of Src (Ab133283) and GAPHD (Ab9485) were purchased from Abcam (Cambridge, United Kingdom). SYBR Green PCR kit (K0223) and BCA protein assay kit (PICPI23223) were purchased from Thermo (Waltham, M.A., United States). JAK enzymes, JAK1, JAK2, JAK3, and Tyk2, were bought from Invitrogen (Carlsbad, CA, United States), Millipore (United Kingdom) Ltd. (London, United Kingdom), Life (Carlsbad, CA, United States), and Invitrogen (Carlsbad, CA, United States), respectively. Ultrafiltration membranes of 30 kDa (YM-30) were purchased from Millipore Co. Ltd. (Bedford, MA, United States). Other reagents, DAPI (C1002), Tunel Kit (11684817910), Trizol (1596-026), and RIPA cell lysate (R0020), were bought from Beyotime Biotechnology (Shanghai, China), Roche Diagnostics Deutschland GmbH (Shanghai, China), Invitrogen (Carlsbad, CA, United States) and Beijing Solarbio Life Sciences and technology Co., Ltd (Beijing, China), respectively. Formic acid, ammonium, methanol, chloroform, dimethyl sulfoxide (DMSO), and other reagents of analytical grade were purchased from Sinopharm Chemical Reagent Co., Ltd. (Shanghai, China).

### Materials

Fresh plant materials of *Z. simulans* were collected from Wuhan Botanical Garden in September 2018, under the plant material collection guidelines. The authentication and identification of the plant were conducted by a professional taxonomist (Assistant Prof. Wenbin Xu) of the gardening center from Wuhan Botanical Garden. A voucher specimen (NO. 2018ZS001) was deposited in the public herbarium of Wuhan Botanical Garden. The bark of the fresh plant was collected and dried at 30°C. After that, the bark material was crushed and stored in a drier till use. As described, the crude alkaloids of *Z. simulans* were prepared and further purified by the optimized WCX solid-phase conditions to get total quaternary alkaloids (QAs) (Tian et al., 2017; Fan et al., 2019). The fifteen samples of QAs were combined and stored at 4°C for further use.

### Affinity Ultrafiltration Screening

The JAKs (10 μg) were dispersed in 1 ml with phosphate buffer (PBS, pH 7.5) and separated into 10 μL per aliquot in EP tubes after being vortexed. The QAs were dissolved by PBS to 2 mg/ml for the following affinity ultrafiltration and marked as QAs-PBS. The inactive JAKs for negative control were boiled for 10 min and conducted affinity ultrafiltration with active JAKs in parallel and triplicate. For affinity ultrafiltration, 300 μL phosphate buffer and 100 μL QAs-PBS were added to mix with active and inactive JAKs aliquots in triplicate, while incubated for 30 min at 37°C. After incubation and filtration through a YM-30 membrane (30 kD) at 10000 rpm for 5 min, the unbounded compounds were washed away by rinsing three times with 400 μL PBS. Then, the ultrafiltration filters were washed with methanol to collect compounds with affinity to JAKs. The eluents of active and inactive JAKs were combined and dried under a nitrogen stream to produce residues, respectively, which were redissolved in 100 μL of 5% methanol solution for the following LC or LC-Q-TOF-MS analysis.

### Identification of Alkaloids With LC-Q-TOF-MS Analysis

A Waters Arc 2998 HPLC with an auto-sampler and a PAD detector was employed for the LC analysis. To achieve peak separation, the chromatography method was developed. Briefly, a 5 μL sample was analyzed on a Waters X Select CSH column (2.5 μm 2.1 × 150 mm) at 30°C. The flow rate was set at 0.2 ml/min and the chromatograms were recorded at 280 nm. Formic acid-H_2_O solution (0.1%, A) and acetonitrile (B) were used as mobile phases, and the gradient was set as follows: 0–5 min, 5% (B); 5–25 min, 5%–25% (B); 25–60 min, 25%–95% (B).

For the MS analysis, an Agilent 1290 HPLC system combined with Agilent 6530 Q-TOF-MS was employed, and the TOF-MS data was collected in full scan between m/z 100 to 1000 in the positive mode. The mass conditions were set as follows: gas temperature, 300°C; drying gas, 8 L/min; nebulizer pressure, 25psi; sheath gas temperature, 350°C; sheath gas flow, 12 L/min; capillary Voltage, 3500 V; fragmentor voltage, 175 V; skimmer voltage, 65 V; OCT 1 RF Vpp, 750 V. Ramped collision energy was set as at 10, 20 and 40 V to obtain MS/MS fragment data. The alkaloids detected were identified based on the comparison of exact molecular weight and MS/MS spectra with standards and reported literatures.

### Anti-Proliferative Activity Evaluation

For the anti-proliferative activity analysis, human LO2 and AGS cell lines were purchased and used in the Sulforhodamine B (SRB) Assay (Ammar et al., 2018). The cells were incubated on a 96-well plate under 5% CO_2_ at 37°C for 24 h. After that, the compounds were dissolved and diluted into a series of concentrations, including Magnoflorine (peak 5), Berberine (peak 19), Chelerythrine (peak 21), and Cerdulatinib. DMSO and Cerdulatinib were used as blank and positive controls, respectively. After chemicals addition and 72 h incubation, the supernatant was discarded, and 50% trichloroacetic acid (TCA) was added into the wells to fix cells for 30 min. After being washed and dried, the cells were stained with 0.04% SRB for 30 min and then washed with 1% acetic acid to remove the unbound dye. The optical density (OD) values were determined at 565 nm. The inhibitory rate equals to (ODC-ODT)/ODC×100%, where ODT and ODC are the OD values of the blank and the screened potential alkaloids, respectively.

### Molecular Docking

For docking studies, molecular docking was performed by using Molecular Operating Environment (MOE v2019.01) on the basis of the reported literatures (Ren et al., 2020; Shu et al., 2020). The structures of compounds, Magnoflorine (peak 5), Berberine (peak 19), Chelerythrine (peak 21), and positive control (Cerdulatinib), were prepared and optimized by energy-minimizing to be used as ligands in the following docking, respectively. The structures of JAKs receptors were obtained from RCSB Protein Data Bank (JAK1 PDB code, 6SM8; JAK2 PDB code, 3KRR; JAK3 PDB code, 5TTS; Tyk2 PDB code, 5C03). After removing water molecules and adding hydrogen atoms, the structures of proteins were subjected to energy minimum conformation. Through the Site Finder tool, active sites in the targeted proteins were provided. Then, the prepared protein structures were used for docking calculation with the optimized candidates in the active sites. The docking scores were calculated by the London dG scoring function through the “Triangle Matcher” method. Docking results were evaluated by interaction energy, bonds, and functional residues in the following discussion.

### Apoptosis Assay

To detect apoptosis cells, the TUNEL method was used in this experiment. Briefly, logarithmic growth cells were inoculated. Chelerythrine with a final concentration at 11.81 μM was added when cells covered 50%–60% of the plates. The cells were incubated for 48 h. After fixing with formaldehyde for 48 h and washing with PBS, the nuclei indicating apoptotic cells were stained and preserved according to the instruction of the TUNEL detection kit. The apoptotic nuclei were stained green to facilitate an examination under a microscope.

### Adhesion Assay

To evaluate the morphology alteration of Chelerythrine, 50%–60% logarithmic growth cells of full glass plates were treated with Chelerythrine (11.81 μM). After 48 h incubation, the cell suspension was collected and added into precoated 12-well plates with FN. After incubation for an additional 1 h, the cancer cells were obtained by discarding supernatant and fixed with 4% paraformaldehyde for 15 min. Crystal violet was used to stain for 30 min to make it easy to observe the morphology variation under a microscope.

### Migration Assay

To investigate the inhibitory effects of Chelerythrine for migration capacity, AGS cells were cultured in culture dishes. Once 80% bottom of the dishes were covered by AGS cells, 10 μL pipettes were employed to clean the cells along the marker perpendicularly. After the necessary wash with PBS, Chelerythrine was added with a final concentration at 11.81 μM for 48 h. A blank was set as a negative control. To observe the potential effects of Chelerythrine on migration, images at 0 and 48 h were recorded under a microscope.

### Invasion Assay

To test invasion capacity, a Transwell invasion assay was conducted in this experiment. The AGS cells at the logarithmic growth phase were inoculated into 6-well plates, and Chelerythrine with a final concentration at 11.81 μM was added when cells reach 50%–60% confluence. Instead of Chelerythrine, DMSO was used as a negative control. After 48 h incubation, the cell suspension was collected and inoculated into Transwell chambers (200 μL of each). 700 μL of complete culture medium containing 10% FBS was added to 24-well plates in the sub-chamber and cultured at 37°C for 48 h. The cells at the bottom of the sub-chamber were fixed by immersing cells in 1000 μL 4% formaldehyde solution for 10 min. The cells were washed in PBS and stained with crystal violet for 30 min. The cells in the Transwell chamber were regarded as cells without migration and cleaned. The invaded cells were visualized and quantitated under a microscope.

### qPCR Analysis

qPCR was performed to explore the potential transformation change of estrogen signal pathway genes which resulted from Chelerythrine using specific primers and the ABI Prism 7300 analyzer (Applied Biosystems, Foster City, CA). Target gene expression values were normalized to human GAPDH. After inoculating logarithmic growth AGS cells in 6-well plates and culturing till 50%–60% of the plates were covered, Chelerythine was added to a final concentration at 11.81 μM. The supernatant was discarded and Trizol was added to extract RNA. The RNA extraction referring to estrogen genes, ER-α36, ER-α66 and ER-β1, was dissolved in diethyl pyrocarbonate, and stored at −80°C. A blank was set as a control to amplify with the sample group, and the fluorescence intensity during the PCR reaction was monitored in real time.

### Western Blot Assay

AGS cells were seeded into a 6-well plate to culture. When 50%–60% bottom were covered, Chelerythrine with a final concentration at 0, 2.95, 5.91, and 11.81 μM were added. After 48 h incubation and supernatant discarding, a 250 μL RIPA tissue cell rapid lysate was added and centrifuged at 4°C for 15 min at 12000 g to obtain supernatant. BAC method was taken to have the total protein concentration determined. The total proteins (20 μg) were isolated by PAGE gel electrophoresis, transferred the protein to polyvinylidene fluoride membrane by wet transfer method, and closed at room temperature for 1 h by 0.5% skimmed milk powder.

A diluted primary antibody was added according to the instructions and stored at 4 °C overnight. Secondary antibody HRP-labeled was added after washing 3 times with TBST and incubated at room temperature for 1 h. After washing another 3 times with TBST, an ECL kit was used to emit light. The expression of Src protein was imaged and analyzed through the biochemical detection system with GAPDH as an internal reference.

### Statistical Analysis

Experiments were conducted in triplicate. For statistical analysis, SPSS 16.0 was used in the independent *t* test analysis. Data were shown as mean value ±SD. A *p* value <0.05 (marked as *) or 0.01 (marked as **) indicated the difference between the two groups was statistically significant.

## Results and Discussion

### Screening of Potential JAKs Inhibitors

To separate and characterize the alkaloids in the following affinity screening, optimizations of chromatography conditions were conducted based on our previous study (Fan et al., 2019), mainly containing different types of chromatographic columns, mobile phases, and elution gradients. Under the newly established chromatographic condition, qualitative and relative quantitative analyses of samples pre-prepared from *Z. simulans* extracts were implemented with LC and LC-Q-TOF. Then, four subtypes of Janus kinases (JAKs), including JAK1, JAK2, JAK3, and Tyk2, were employed to explore the potential and selective JAK inhibitors. More stable alkaloids-enzyme complexes suggested stronger anti-proliferative bio-activity, which were reflected by affinity to diverse JAKs. In order to exclude the interference of physical absorption, enrichment factors (EFs) were introduced in this study to evaluate and visualize the affinities between compounds and JAKs (Chen et al., 2020). The active and inactive JAKs enzyme samples were analyzed and recorded under the optimized condition in parallel. EFs = (As-Ac)/A_0_×100%, where As, Ac, and A_0_ represent the peak areas of active, inactive JAKs samples and QAs, respectively.

**FIGURE 1:**
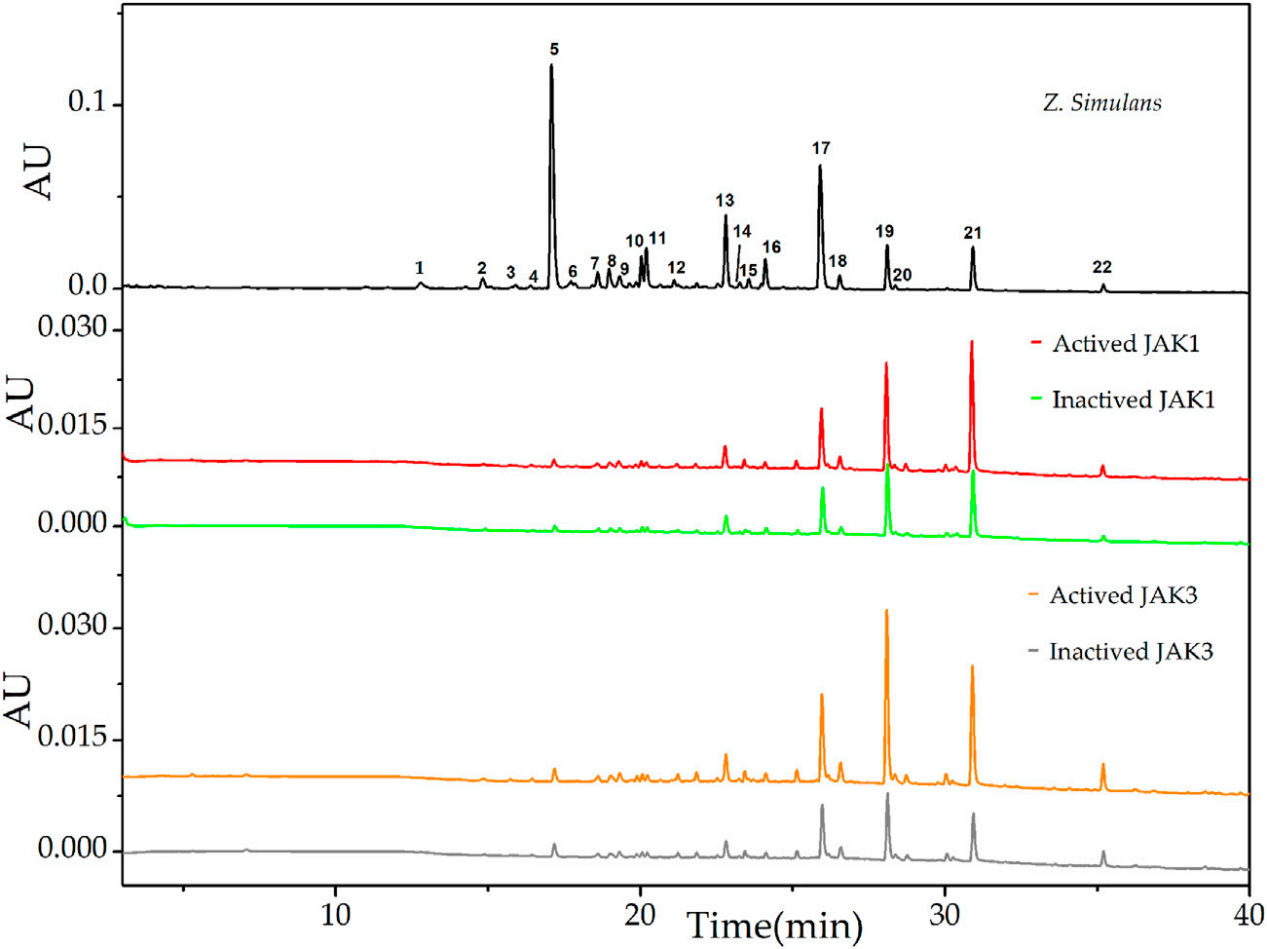
UF-LC chromatograms of the potential ligands from total alkaloids of *Z. simulans* for JAK1 and JAK3. Inactive JAK1 and JAK3 were produced by a pre-boiled water bath and conducted ultrafiltration in parallel with active JAKs. Different colors, red and green, were used to indicate active and inactive JAK1 treatment, while orange and gray for active and inactive JAK3, respectively.

After affinity ultrafiltration analysis (UF-LC-TOF) and EFs calculation, the results were shown in Table 1. It is reported that the four conserved domains of JAKs were composed of seven homology domains (JH) (Huang et al., 2019). Due to the structural homology of targets employed in ultrafiltration (Huang et al., 2019), the detected peaks from chromatograms may show some similarity. However, EFs still provided evidence of discrepancy enrichment for different kinases. Taking the specificity of the JAK1/3 complex only activated by cytokines containing γ chain and clinic adaption into consideration (Huang et al., 2019), UF-LC chromatograms of JAK1 and JAK3 were presented in Figure 1. For JAK1, peak 21 showed the highest EFs of 41.71%, followed by peak 15 of 26.44%, peak 22 of 24.94%, peak 19 of 22.62%, and peak18 of 10.57%, and other peaks of EFs below 10.0%. For JAK3, peak 19 showed the highest EFs of 59.63%, followed by peak 21 of 40.73%, peak22 of 40.80%, peak 20 of 39.78%, peak18 of 15.45%, and other peaks below 11.0%.

**TABLE 1:** The EFs, RPA ,and identification of the potential ligands for JAKs in QAs.

Peak NO.	Rt (min)	M^+^ (m/z)	MS/MS spectrum	EFs (%)				RPA (%)	Identification
				JAK1	JAK2	JAK3	Tyk2		
1	12.79	274.1463	256.1400, 241.1096, 226.0879, 209.0875, 202.0871, 188.0723, 172.0770, 144.0826, 128.0485	0.00	0.00	0.00	0.00	1.45	2H-pyrano [2,3-b]quinolinium or its isomers Qing et al. (2014b)
2	14.83	286.1464	269.1188, 255.0933, 237.0946, 228.1077, 219.0777, 210.1037, 175.0774, 160.0502, 151.0754, 145.0675, 137.0605, 107.0505	0.00	0.00	1.01	0.00	1.70	7-Methoxylhigenamine Blunt et al. (2017)
3	15.91	344.1888	269.0762, 253.0769, 217.0694, 207.0844, 192.1039, 143.0506, 137.0614	0.00	0.00	0.00	0.77	0.86	Isotembetarine or xylopinidine Tian et al. (2017)
4	16.39	328.1569	312.1155, 278,1770, 254.1101, 239.1210, 178.0881, 163.0650, 149.0510, 134.0632	4.57	13.22	8.64	1.25	0.49	Unidentified
5	17.09	342.1729	311.1326, 297.1187, 282.0899, 265.1874, 251.0738, 222.0693, 219.0811, 207.0818, 191.0868	0.22	0.53	0.038	0.25	33.84	Magnoflorine^a^ Nikolic et al. (2012)
6	17.72	312.1262	297.1038, 282.0806, 268.0985, 253.1100, 240.1035, 224.1052, 213.1126, 177.0794, 160.0843, 152.0767, 148.0767, 133.0497	0.00	0.00	0.00	0.457	1.71	Unidentified
7	18.60	330.1730	299.0316, 207.0809, 192.1043, 177.0807, 175.0783, 151.0756, 143.0490, 137.0613	1.70	10.25	5.14	3.63	2.12	Reticuline Nikolic et al. (2012)
8	18.98	358.2042	313.1472, 239.1078, 221.10474, 206.1204, 192.1040, 189.0925, 174.0703, 163.0778, 158.0742, 151.0758, 137.0607	7.12	11.51	5.76	8.63	2.54	8-Methoxy-isotembetatrine Tian et al. (2017)
9	19.32	314.1775	269.1191, 239.1003, 209.0972, 192.1028, 183.0769, 175.0715, 165.1047, 160.0498, 143.0499, 137.0589, 115.0554, 107.0511	6.57	12.03	5.23	4.74	1.97	Magnocurarine or its isomers Nikolic et al. (2012); Shu et al. (2020)
10	20.04	356.1885	192.1035, 177.0793, 148.0776	0.59	6.36	2.02	2.15	3.13	N-Methyltetrahydrocolumbamine Nikolic et al. (2012)
11	20.20	356.1855	311.1221, 296.0825, 279.1101, 265.0860, 253.0870, 248.0842, 236.0843, 219.0844, 207.0823, 191.0867, 178.0783	0.40	2.21	1.02	1.31	4.79	Menisperine Nikolic et al. (2012)
12	21.11	304.1569	286.1424, 271.1181, 262.1058, 256.0974, 246.1148, 232.1003, 223.1042, 206.0854, 202.0858, 072.0767	2.47	6.53	1.42	1.35	0.96	Unidentifed
13	22.81	356.1885	347.8599, 340.1579, 326.1421, 296.1071, 281.0841, 265.0864, 253.0882, 237.0938, 225.0871, 210.0667, 192.1047, 177.1047	2.25	12.79	3.46	3.28	8.20	Xanthoplanine Nikolic et al. (2012)
14	23.26	368.1523	352.1195, 338.1103, 336.1241, 324.1230, 310.1118, 306.0803, 292.1034, 278.0826	6.77	9.72	3.017	6.34	0.64	10-Hydroxy-2,3,9,12-tetramethoxy-jatrorrhizine Tian et al. (2017)
15	23.56	354.1731	190.0885, 175.0643, 160.0779	26.44	7.343	11.00	0.59	1.00	N-methylcanadine or its isomers Qing et al. (2014a)
16	24.11	342.2098	297.1509, 282.1247, 266.1193, 251.1106, 237.0931, 223.0733, 189.0908, 176.0809, 163.0787, 121.0656	0.911	6.85	2.31	2.49	3.42	Unidentifed
17	25.91	354.1729	338.1434, 324.1261, 306.1150, 292.1175, 190.0879, 175.0879, 165.0922, 149.0605, 135.0445	2.75	14.15	5.58	4.51	17.96	N-methylcanadine or its isomers Qing et al. (2014a)
18	26.55	354.1730	207.0873, 190.0875, 159.0440, 149.0602, 131.0501	10.57	24.40	15.45	4.41	1.57	N-methylcanadine or its isomers Qing et al. (2014a)
19	28.37	336.1256	320.0924, 306.0786, 304.0995, 292.0973, 278.0833, 263.0887	22.62	73.82	59.63	10.40	5.04	Berberine^a^ Qing et al. (2014a)
20	28.11	334.1101	319.0871, 304.0620, 291.0904, 276.0679, 262.0895	8.92	38.56	39.78	10.56	0.56	Isoterihanine Qing et al. (2014a)
21	30.92	348.1255	332.0940, 330.0787, 318,0768, 304.0986, 209.0830, 287.0970	41.71	56.31	40.73	16.73	5.13	Chelerythrine^a^ Tian et al. (2017)
22	35.20	318.3030	300.2955, 260.8465, 256.2656, 220.7547, 132.1029, 102.0914	24.94	34.53	40.80	13.67	0.83	Unidentifed

RPA, relative peak area.

a Identified by comparing with corresponding standards.

**FIGURE 2:**
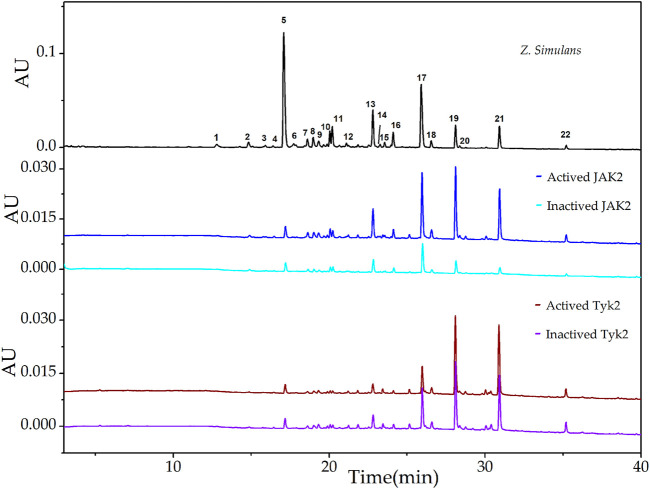
UF-LC chromatograms of the potential ligands from total alkaloids of *Z. simulans* for JAK2 and Tyk2. These two JAKs were boiled to get inactive kinases and conducted ultrafiltration in parallel. The colors, blue and cyan, were employed to present active and inactive JAK2 treatment, while wine and violet for active and inactive JAK3, respectively.

The downstream signal pathway may be mediated by IL-10 family cytokines and glycoprotein 130 (Huang et al., 2019), UF-LC chromatograms of JAK2 and Tyk2 were presented in Figure 2. For JAK2, peak 19 showed the highest EFs of 73.82%, followed by peak 21 of 56.31%, peak 20 of 38.56%, peak 22 of 34.53%, peak 18 of 24.40%, and other peaks below 15.00%. For Tyk2, peak 21 showed the highest EFs of 16.73%, followed by peak 22 of 13.67%, peak20 of 10.56%, peak 19 of 10.40%, and peak 8 of 8.63%, and other peaks below 6.34%.

The results of EFs suggested that compounds corresponding to peak 19, peak 20, peak 21, and peak 22 possess a strong affinity to JAK kinases. In addition, these four compounds displayed higher affinity degrees to JAK1, JAK2, and JAK3 than Tyk2. Compared with peak 20 and peak 22, peak 19 and peak 21 were the most potent active inhibitors in terms of the relative content of peak areas in the QAs chromatogram. Contrary to the most intensity in QAs alkaloids, peak 5 displayed almost no affinity to JAKs, with the EFs of JAK1 at 0.22%, JAK2 at 0.53%, and JAK3 at 0.038%, Tyk2 at 0.25%, respectively. Thus, it could be used as the negative control in the following study. As a result, based on alkaloids identified from QAs and their corresponding EF values, the rough affinities of QAs to the four subtypes of JAKs were assessed.

### Structural Identification

**FIGURE 3:**
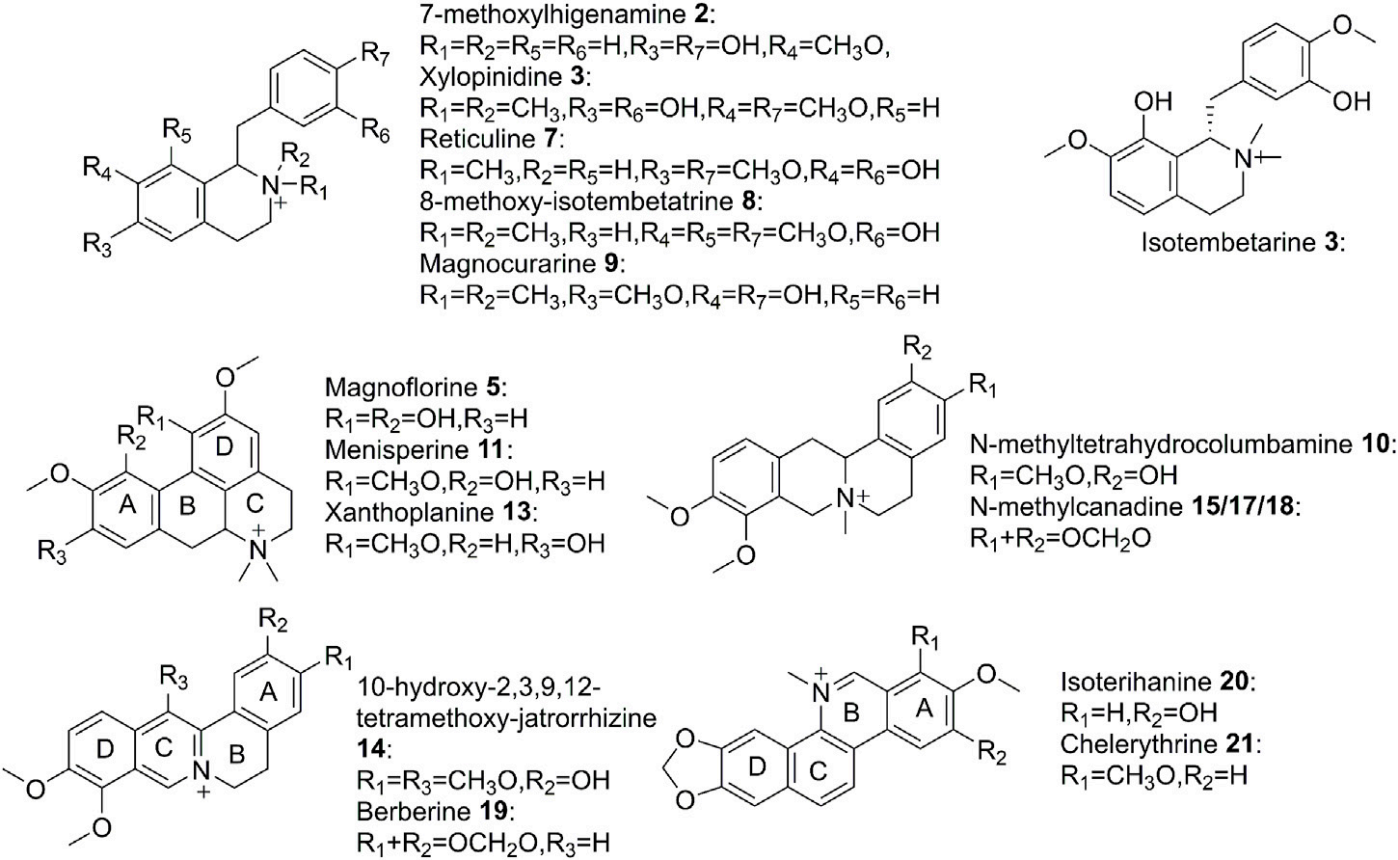
Structures of alkaloids detected in QAs.

To figure out the potential bioactive inhibitors of JAKs, total QAs from *Z. simulans* were separated under the optimized chromatography condition by LC and LC-Q-TOF in the positive mode. As a result, 22 peaks of alkaloids in this sample were recorded, 17 of which were identified based on the comparison of MS/MS fragments with the reported literatures and our previous study (Dai et al., 2012; Nikolic et al., 2012; Jia et al., 2013; Qing et al., 2014a; Qing et al., 2014b; Tian et al., 2017). The structures of the 22 detected alkaloids were shown in Table 1 and Figure 3. For two peaks (peak 19 and peak 21) with the most potential affinity to JAKs, the identification of these two peaks as Berberine and Chelerythrine were further validated by comparing the retention times, quasi-molecular ions ([M]^+^), and MS/MS fragments with their correlated chemical standards, respectively.

### Anti-Proliferative Activity Evaluation

To verify the results of affinity screening, compounds of peak 5 (Magnoflorine), peak 19 (Berberine) and peak 21 (Chelerythrine), were used for the anti-proliferative activity. Reported as a strong inhibitor of JAKs, Cerdulatinib was selected as a positive control (Blunt et al., 2017). For cell lines, LO2 and AGS were employed and treated with the compounds, which were dissolved with DMSO and diluted into a series of concentrations. As presented in Table 2, Chelerythrine and Berberine exhibited obvious anti-proliferative potential. For LO2, Chelerythrine and berberine showed remarkable cell inhibition with IC_50_ at 4.51 and 24.20 μM, respectively, compared with the IC_50_ of Cerdulatinib at 11.38 μM. For AGS, Chelerythrine and Berberine showed significant cell proliferative inhibition with IC_50_ at 11.81 μM and 74.86 ± 0.37 μM, respectively, while IC_50_ at 28.89 ± 2.42 μM for Cerdulatinib. Nevertheless, no obvious inhibitory effects of Magnoflorine on LO2 and AGS were observed. The inhibition of peak 19 (Berberine) and peak 21 (Chelerythrine) were more significant to LO2 over AGS, which may lead to some liver toxicity since LO2 and AGS are derived from normal hepatic cells and gastric adenocarcinoma cells, respectively. Consequently, the further ligand-target interaction figuration may pave the way for further structure modification and future exploration to produce strong but selective pro-drug.

**TABLE 2:** The IC50, and docking results of selected potential ligands.

No.	Compounds	AGS	LO2	JAK1	JAK2	JAK3	Tyk2
		(IC_50_ μM)	(IC_50_ μM)	Residues with interaction (ES, kcal/mol)	Residues with interaction (ES, kcal/mol)	Residues with interaction (ES, kcal/mol)	Residues with interaction (ES, kcal/mol)
1	Chelerythrine	7.17	4.51	Asp1021 (-2.3)	Leu855 (-2.7)	Met902 (-0.5) Leu828 (-2.3)	Val603 (-2.0)
					Gly856 (-0.7)		
2	Berberine	74.86 ± 0.37	24.20	Asp1021 (-3.3)	Arg980 (-3.0)	Leu828 (-3.8)	Pro694 (-0.9)
					Leu855 (-0.6)		
3	Magnoflorine	-	-	Glu966 (-0.8)	Arg980 (-0.8)	Leu828 (-0.5)	Val603 (-0.5)
				Leu1010 (-0.5)	Leu855 (-0.5)		
4	Cerdulatinib^a^	28.89 ± 2.42	11.38	Arg879 (-5.0)	Leu932 (-2.2)	Leu905 (-2.3)	Asp696 (-0.6)
				Lys908 (-1.9)	Met929 (-2.4)	Arg911 (-3.4)	Arg739 (-0.7)
				Gly882 (-1.4)	Gln854 (-1.4)	Leu828 (-1.6)	

-, not detected; ES, energy scores.

a Positive control.

### Molecular Docking Analysis

**FIGURE 4:**
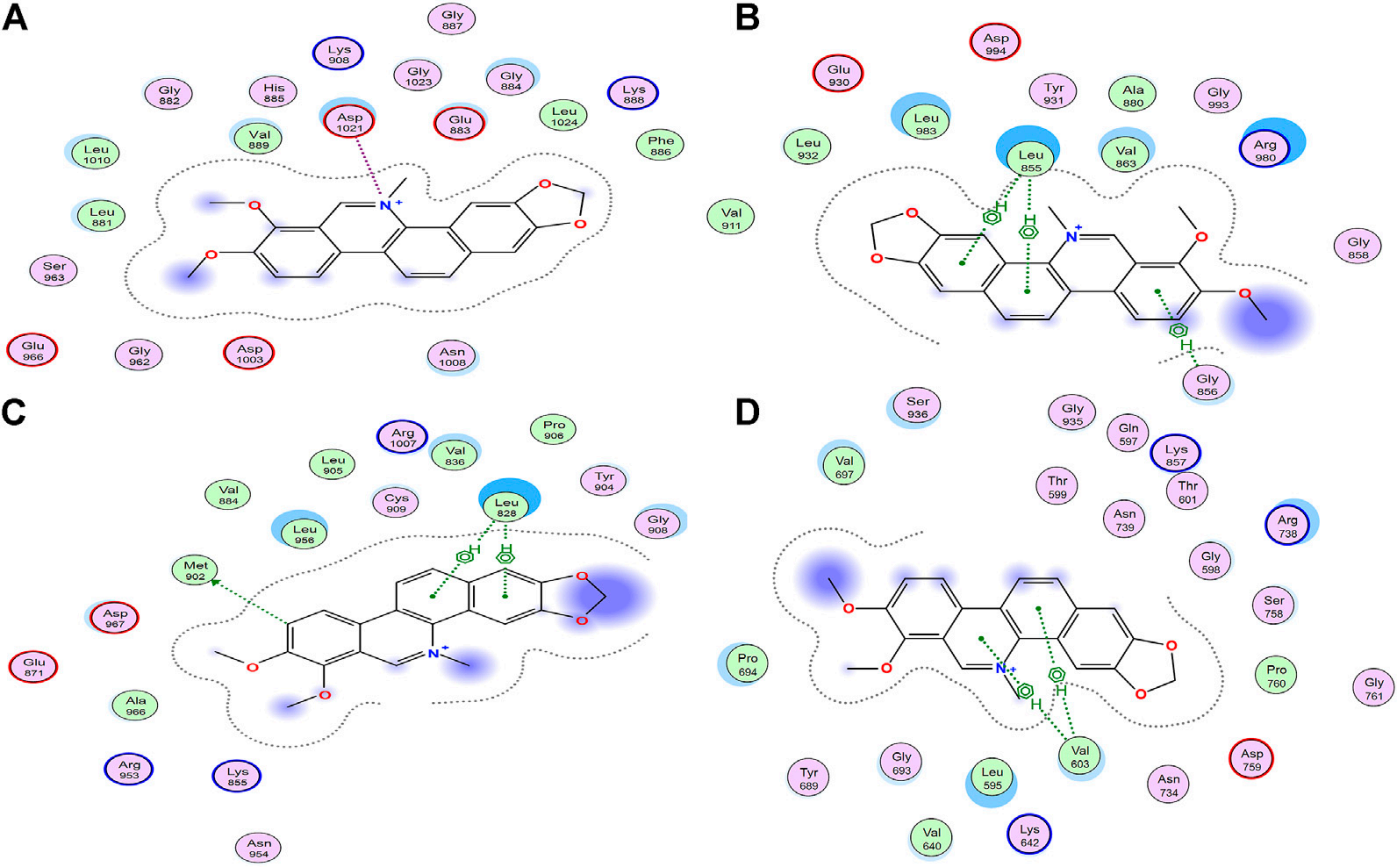
Interactions between Chelerythrine and JAKs. Molecular docking was used to display the interactions between Chelerythrine and **(A)** JAK1, **(B)** JAK2, **(C)** JAK3, and **(D)** Tyk2, respectively. Original structures of JAKs receptors were downloaded from RCSB Protein Data Bank (JAK1 PDB: 6SM8; JAK2 PDB: 3KRR; JAK3 PDB: 5TTS; Tyk2 PDB: 5C03).

Molecular docking has been used to guide chemical structure modification and illustrate the ligand-target interactions (Işık et al., 2020). By simulating calculation, molecular docking can provide insight into the mechanism between potential ligands and targeted macromolecular receptors, and provide details on targeted amino acid residues, interaction bonds, binding energy, and action pocket (Işık et al., 2020). To investigate the mechanism of inhibitory activities above, Molecular Operating Environment (MOE) software was employed. As shown in Table 2, the distinguished interactions between compounds and targets were observed as follows. To better understand the docking simulations in this study, the interactions between Chelerythrine and JAKs were presented in Figure 4.

It was well-studied that active ligands form certain bonds with the amino acid residues of targets, mainly including ionic bonds, hydrogen bonds (H-bonds), van der Waals force, and hydrophobic interactions (Chen et al., 2020). In contrast to H-bonds and hydrophobic interaction for JAK2, JAK3, and Tyk2, ionic bonds were observed for JAK1. To present the distinct interactions, Energy Scores (ES) were employed to display the intensity of the ligand-enzyme complexes. For JAK1, peak 19 (Berberine) and peak 21 (Chelerythrine, Figure 4A) only formed ionic bonds with Asp1021 at the quaternary ammonium N molecular with the energy at −3.3 kcal/mol and −2.3 kcal/mol, respectively, while peak 5 (Magnoflorine) formed weaker ionic bond and H-bonds with Glu966 and Leu1010, along with energy at −0.8 kcal/mol and −0.5 kcal/mol, respectively. Unlike peaks 5, 19, and 21, the significant interaction (total ES at −8.3 kcal/mol) between Cedulatinib and JAK1 owed to the H-bonds with residues of Gly822, Lys908, and Arg879.

For JAK2, the docking sites with the four ligands were primarily on the amino acid residues, such as Leu855, Gly856, Arg980, Leu932, Met929, and Gln854. Except for Cerdulatinib, the other three alkaloids formed three common H-bonds with Leu855 at −2.7 kcal/mol for Chelerythrine, −0.6 kcal/mol for Berberine, and −0.5 kcal/mol for Magnoflorine, respectively. Moreover, the ES between the A ring of Chelerythrine and Gly856 was −0.7 kcal/mol (Figure 4B). Arg980 of this enzyme interacted with epoxy atoms from the D ring of Berberine (−3.0 kcal/mol) and N methyl from the C ring of Magnoflorine (−0.8 kcal/mol). As for positive control (Cerdulatinib), H-bonds were formed due to the interactions with Leu932, Met929, and Gln854, and resulted in higher affinity with ES at −6.0 kcal/mol.

For JAK3, the residues that participated in docking included Met902, Leu905, Arg911, and Leu828. Similar to JAK2, Leu828 acted as common amino acid residue but with distinguished pharmacophore and ES, such as C and D rings of Chelerythrine (−2.3 kcal/mol) (Figure 4C), C and D rings of Berberine (−3.8 kcal/mol), A ring of Magnoflorine (−0.5 kcal/mol), and N polycyclic ring of Cerdulatinib (−1.6 kcal/mol), respectively. Compared with the H-bonds between the A ring of Chelrythrine and Met902 (−0.5 kcal/mol), the interactions between the O atom of Cerdulatinib and Leu905 (−2.3 kcal/mol) and Arg911 (−3.4 kcal/mol) was significantly stronger.

As to Tyk2, the four ligands formed H-bonds with residues Val603, Pro694, Asp696, and Arg739. Val603 interacted with Chelerythrine (Figure 4D) and Magnoflorine with ES at −2.0 kcal/mol and −0.5 kcal/mol, respectively, while Pro694 formed H-bonds with the C ring of Berberine with ES at −0.9 kcal/mol. Compared with the three alkaloids, Cerdulatinib showed the potential for interaction with Arg738 (−0.7 kcal/mol) and Asp696 (−0.6 kcal/mol) in Tyk2.

Our results suggested that JAK1, JAK2, and JAK3 might be important targets and play vital roles in the inhibition of LO2 and AGS cell proliferation (Shi et al., 2019; Wang et al., 2019). In addition, amino acid residues, such as Leu855 in JAK2 (Wang et al., 2019), Asp1021 in JAK1 (Kim et al., 2015), and Leu828 in JAK3 (Yu et al., 2019), were the common binding sites of JAKs active pocket, and docking simulations were conducted in the ATP binding sites of JAKs. Different from compounds with polar moieties to exhibit three-dimension configuration, higher docking energy and more interaction sites with JAKs (Yu et al., 2019; Ren et al., 2020), such as Cerdulatinib used in this study, the structures of alkaloids from QAs seemed to have more rigidity, less polarity, and more hydrophobicity. As to alkaloids from QAs, Chelerythrine and Berberine showed higher docking energy with JAKs than Magnoflorine. Yet, the same characteristic interactions between ligands and amino acid residues were observed, e.g., Leu828 in JAK3 with these three alkaloids. However, the Quaternary N atom seems to be in a plane with an adjacent ring for Chelerythrine and Berberine, while not for Magnoflorine. The distinguished bioactivity and interaction energy may be attributed to different structures in spatial to some extent. Moreover, quaternary N atom and hydrophobic rings in QAs might be the hinge-region binding motifs, which could contribute to affinity binding between the alkaloids and the ATP binding sites in JAKs.

### Effects on AGS Cell Apoptosis, Adhesion, Migration and Invasion

**FIGURE 5:**
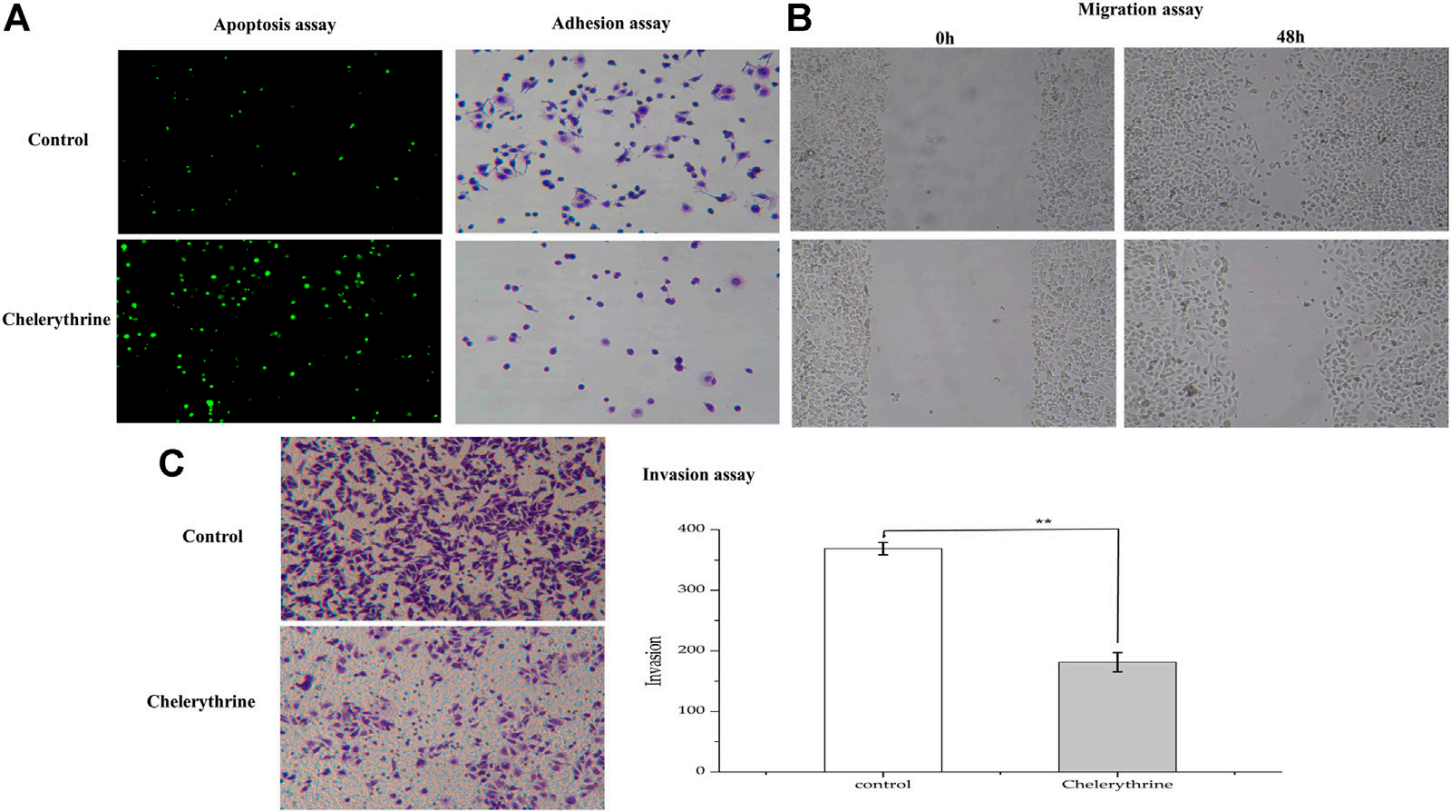
Anti-proliferative effects of Chelerythrine on Gastric cancer Cells (AGS). AGS were treated with 11.81μΜ Chelerythrine for 48 h to explore its effects on adhesion, migration, invasion, and apoptosis (*n* = 3). DMSO was used as negative control (Control). **(A)** Apoptosis and Adhesion assay. **(B)** Migration assay. Images at 0 and 48 h were recorded for Chelerythrine treatment and negative control groups. **(C)** Invasion assay. Images (left) and counts (right) of Transwell cells. **: *p* < 0.01.

As the most anti-proliferative alkaloid in this study, Chelerythrine was regarded as a good candidate for contributing cancer cells apoptotic inhibitory to various cancer cells (Malikova et al., 2006), such as HCC (Hepatocellular cancer cells), Hela (Cervical cancer cells), DU145 (Prostate cancer cells) (Zhu et al., 2018; Binbin Yang et al., 2020; Tianfeng Yang et al., 2020). Due to the lack of comprehensive characterization of the effects of Chelerythrine on gastric cancer cells, the morphology change study of AGS is needed to further understand the inhibitory effects. In order to investigate the function of Chelerythrine on promoting or inhibiting AGS cells, Chelerythrine at 11.81 μM was employed in the *in vitro* assays. The effects of Chelerythrine on gastric cancer cells apoptosis, adhesion, migration, and invasion were shown in Figure 5.

To investigate the effects of Chelerythrine on apoptosis, a sensitive detection method, the TUNEL method, was employed. Apoptotic nuclei of cancer cells were labeled *in situ* by the TUNEL method, and stained green when observed under a microscope (Yang et al., 2017). As shown in Figure 5A, the fluorescence intensity for Chelerythrine treated AGS cells is stronger than the untreated group (*p* < 0.01), which indicated more apoptosis resulting from Chelerythrine. For cancer cells, it is well known that cytoskeleton is associated with carcinoma cells adhesion and motility (Farmer et al., 2019), we assessed whether Chelerythrine alters AGS morphology cytoskeleton. After 48 h incubation, negative group cells displayed elliptical and growing adhesion to well. In contrast, cells treated with Chelerythrine displayed more rounded cells and less adhesion. In addition, the figures of migration in Figure 5B exhibited AGS cell growth at 0 and 48 h. After 48 h incubation, cells treated with Chelerythrine showed significantly decreased viability, while untreated almost covered the pre-marked blank space. Viability and adhesion ability may contribute to malignant cancer cell invasion, which further leads to distant metastasis of various organs (Hao and Yang, 2019). As shown in Figure 5C, treatment of AGS cells with Chelerythrine significantly decreased the invasion of AGS cells by comparing the counts of Transwell cells with untreated cells (*p* < 0.01). By evaluating the effects on AGS in this part, the results revealed Chelerythrine effects on promoting apoptosis, weakening adhesion, decreasing migration, and inhibiting invasion.

### Regulation on ER-α36, ER-α66, ER-β1 and Src

Based on ultrafiltration of four JAKs, Chelerythrine was proposed as the most potent anti-proliferative chemical in QAs. As an upstream activator, Src was reported to mediate the biological processes by regulating the JAK-STAT signal pathway, including proliferation, apoptosis, adhesion, migration, and invasion (Yan et al., 2017). Owing to the attention brought by hormones related to gender in breast cancer (Chlebowski et al., 2020), estrogen and its corresponding effects on cancer cells were investigated during various carcinoma cells (Deli et al., 2019). In addition, conventionally risks referring to infection, environmental risk, and diet, the incidence, and prevalence of gastric cancer between men and women accounted for significant differences (Wang et al., 2013). It is reported that ER-α36 mediated the estrogen signal pathway to stimulate proliferation, adhesion, migration, and invasion of gastric cancer cells by activating the Src signal pathway (Wang et al., 2013). In addition, ER-α66 expression was associated with diffuse-type gastric cancer and shorter disease-free survival, and the presence of ER-β might provide protective effects on inhibiting invasiveness for gastric cancer cells (Saif and Jiang, 2016). Thus, Chelerythrine targeting JAKs may mediate the biological progression of gastric cancer cells (AGS) through regulating estrogen receptors/Src. To explore the potential mechanism, ER-α36, ER-α66, and ER-β1 were determined by qPCR besides Src by Western blot.

**FIGURE 6:**
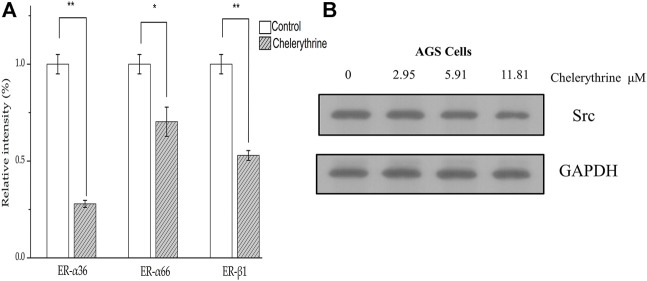
Regulation on estrogen pathway. **(A)**Treated with Chelerythrine at 11.81 μΜ or control for 48 h, mRNA levels of AGS were determined by quantitative PCR. The results were displayed as mean ± SD (*n* = 3). *p* values, *: *p* < 0.05, **:*p* < 0.01. **(B)** Western blot of Src and GAPDH (internal reference) presented after being treated with Chelerythrine at 0, 2.95, 5.91, and 11.81 μΜ.

As shown in Figure 6A, three genes in the groups treated with Chelerythrine at 11.81 μΜ were significantly down-regulated compared with untreated AGS cells. ER-α36 showed most significant down-regulation (*p* < 0.01), followed by ER-β1 (*p* < 0.01) and ER-α66 (*p* < 0.05). Based on the functional effects illustrated earlier, our results suggested that Chelerythrine is likely to enhance the inhibition of AGS cancer cells by down-regulating ER-α36. Meanwhile, the survival might be improved by attenuating the expression of ER-α66. Moreover, Chelerythrine may lead to the protective effects of ER-β1 reduced not only because of the down-regulation but also because ER-β1 is just partly involved in gastric carcinogenesis and limited significance (Saif and Jiang, 2016). The further results of Western blot showed that Src decreased significantly with the increase of Chelerythrine (*p* < 0.05) (Figure 6B). Taken together, our evidence supported that one way Chelerythrine inhibiting AGS down-regulates the ER-α36-Src pathway besides ER-α66 and ER-β1.

## Conclusion

In this study, an ultrafiltration affinity strategy targeting JAKs was successfully established to screen out two potential bio-active alkaloids, Berberine and Chelerythrine, from QAs, which helped with the understanding of JAKs as the anti-proliferative targets on AGS cells. The inhibitory results of LO2 revealed the potential of liver toxicity from Berberine and Chelerythrine. For the two candidates, molecular docking results suggested the ion bond in alkaloids-JAK1 complex and selectivity to JAK1, JAK2, and JAK3 over Tyk2. The discrepancies in interaction bonds and affinity intensities supported Quaternary ammonium N and polar groups as effective moieties in reducing toxicity and enhancing efficacy. As the most anti-proliferative alkaloid in this study, Chelerythrine not only promotes apoptosis but also inhibits adhesion, migration, and invasion by significantly down-regulating ER/Src signal pathway.

In conclusion, on the one hand, the results in this study provided the first piece of evidence that JAKs was one of the major targets for the anti-proliferative activity; on the other hand, the established affinity ultrafiltration LC-Q-TOF-MS method was effective to discover the potential bioactive alkaloids and to better explain its action mechanisms in a multi-component and multi-target manner. In addition, it was the first report that Chelerythrine promoted apoptosis and inhibited proliferation by regulating the expression of estrogen receptors in gastric cancer cells, highlighting the potential use of JAK inhibitors as therapeutic candidates by mediating estrogen receptors.

## Data Availability

The raw data supporting the conclusion of this article will be made available by the authors, without undue reservation.

## Abbreviations

ERs, estrogen receptors; EFs, enrichment factors; H-bonds, hydrogen bonds; ES, energy scores; QAs, total quaternary alkaloids; JAKs, Janus kinases; STAT, signal transducer and activator of transcription; TCM, traditional Chinese medicine.
